# Relationship Between Short Sleep, Exercise Frequency and Media Use with Oral Health in Korean Elementary School Children: A Cross-Sectional Study

**DOI:** 10.3390/children12101399

**Published:** 2025-10-17

**Authors:** Chae-Eun Kim, So-Youn An

**Affiliations:** Department of Dentistry, Graduate School of Wonkwang University, Iksan 54538, Republic of Korea; rlacodms_1@wku.ac.kr

**Keywords:** sleep, lifestyle, oral health, dental caries, periodontal diseases, oral hygiene, malocclusion

## Abstract

**Highlights:**

**What are the main findings?**
  - Short sleep duration, low exercise frequency, and excessive media use were significantly associated with poor oral health in Korean elementary school children.
  - Dental caries prevalence and periodontal disease were strongly related to insufficient sleep.
**What is the implication of the main finding?**
  - Improving sleep duration and healthy lifestyle habits should be prioritized to prevent oral diseases in children.
  - Pediatric dentists can use these findings to guide preventive strategies and parental education.

**Abstract:**

**Background:** Dental caries, periodontal disease, and malocclusion are common childhood oral diseases strongly influenced by lifestyle factors, including sleep, exercise, and media use. In Korea, the prevalence of dental caries among elementary school children is approximately 20–25%, periodontal disease 1–2%, and malocclusion 12–18%. Sleep is a key determinant of child health; insufficient sleep is linked to weakened immunity, higher systemic inflammation, and greater susceptibility to cariogenic bacteria, suggesting a potential pathway to poor oral health. This study aimed to analyze the combined effects of sleep duration, exercise frequency, and media use on oral health indicators in Korean elementary school students. **Methods**: We analyzed Student Health Examination data from the Ministry of Education (2021–2023) for 93,220 children aged 6–12 years. Oral health indicators included dental caries prevalence (DCP), required rate of improved oral hygiene (RRIOH), periodontal disease prevalence (PDP), and malocclusion prevalence (MP). Sleep duration was categorized as short (<8 h) or adequate (≥8 h). Exercise (≥3 times/week) and media use (>2 h/day) were assessed as lifestyle factors. Associations were examined using the Rao-Scott χ^2^ test and logistic regression. **Results**: Short sleep was significantly associated with a higher prevalence of all oral health indicators, with particularly strong associations for DCP and PDP. Low exercise frequency and excessive media use were also linked to increased DCP and RRIOH. These lifestyle factors were closely interrelated with sleep duration. **Conclusions**: Short sleep, infrequent exercise, and high media use form a lifestyle pattern associated with poor oral health in children. Improving sleep and lifestyle habits should be emphasized as a preventive strategy in pediatric dentistry.

## 1. Introduction

Oral disease is one of the most common health problems worldwide and is a major public health issue as it not only reduces the quality of life but also imposes significant social and economic burdens [1]. Dental caries, periodontal disease, and malocclusions are frequent oral diseases among the pediatric population [2]. The risk factors for these three conditions include a wide range of elements such as host-related factors, socioeconomic factors, psychosocial factors, and lifestyle factors [3,4]. In Korea, the prevalence of dental caries among elementary school children has been reported to be approximately 20–25%, periodontal disease around 1.0–2.0%, and malocclusion 12–18%, indicating that these conditions remain important public health concerns in this population [5,6].

Sleep maintains normal brain function and plays an essential role in regulating various physiological systems, and its importance is especially emphasized in children’s health [7,8]. Short sleep duration is associated with childhood development [9] and cardiometabolic [10] health outcomes. Healthy sleep includes not only sufficient sleep duration but also appropriate sleep timing, regular sleep patterns, the absence of sleep disorders, and good sleep quality [11]. Insufficient sleep is related to decreased immunity, increased inflammation, and susceptibility to bacterial infections [8].

According to the Korea Institute for Youth Policy, the average sleep duration of elementary, middle, and high school students in Korea is 7 h and 18 min, which is more than one hour less than the OECD countries’ average sleep duration of 8 h and 22 min. The average sleep duration of elementary school students was 8 h and 41 min. The main causes of students’ sleep insufficiency were academic factors, such as studying, private academies, and extracurricular activities, followed by media use, such as playing games, visiting internet sites, chatting, watching dramas and movies, and listening to music [12].

The National Sleep Foundation recommends a sleep duration of 9–11 h for school-aged children (6–13 years), and 7–8 h or up to 12 h can also be included in the recommended sleep duration range. Insufficient sleep is defined as fewer than 7 h, and excessive sleep as more than 12 h [13]. The average sleep duration of Korean elementary school students reported above does not meet the recommended sleep duration.

Recent studies have focused on the relationship between sleep and oral health. Short sleep duration or late bedtime may increase susceptibility to cariogenic bacteria, thereby increasing the risk factors for dental caries [14]. In addition, sleep regulates the circadian rhythm, and the circadian rhythm affects the formation of dental plaque [15]. Plaque acts as a major defense mechanism that neutralizes substances that cause dental decay. However, sleep insufficiency disrupts the circadian rhythm, reduces salivary secretions, and, as a result, increases the risk of dental caries [15,16].

Furthermore, sleep insufficiency also affects immune function and may be associated with the onset of periodontitis through biological pathways that induce inflammatory responses. Changes in sleep quality and duration can alter levels of inflammatory biomarkers such as C-reactive protein, tumor necrosis factor-α, and interleukin-6 [17,18]. Such inflammatory cytokines are also involved in the development of periodontitis [19,20]. Therefore, sleep insufficiency may be related not only to dental caries but also to an increased risk of periodontitis.

The relationship between exercise and oral health has gained increasing attention in recent years. Regular exercise has been associated with improved systemic health, which indirectly contributes to better oral health outcomes through enhanced immune function, reduced systemic inflammation, and healthier lifestyle patterns. Conversely, insufficient exercise has been linked to an increased risk of metabolic and inflammatory conditions, which may exacerbate susceptibility to oral diseases such as periodontal disease and dental caries [21,22,23].

Media use, particularly prolonged screen time including television viewing and gaming, has been reported to negatively affect oral health. Extended media use is often associated with irregular sleep schedules, increased snacking or consumption of sugar-containing foods, and reduced time allocated for exercise, all of which are recognized risk factors for dental caries and periodontal problems. Moreover, sedentary behaviors tied to excessive media exposure may further compromise overall health and indirectly influence oral health status [24].

These three lifestyle factors—sleep, exercise, and media use—are closely interrelated and may act synergistically in determining oral health outcomes [23]. For example, short sleep duration is frequently associated with low levels of exercise and high media exposure, creating a lifestyle pattern that amplifies risk factors for poor oral health. This underscores the need for integrated prevention strategies.

Thus, the purpose of this study was to evaluate the impact of sleep duration, exercise, and media use on oral health in Korean elementary school students.

## 2. Materials and Methods

### 2.1. Participants

This study was a cross-sectional study using secondary data from the Student Health Examination conducted by the Korean Ministry of Education between 2021 and 2023. The target population was Korean elementary school students aged 6–12 years. Of the total 105,428 subjects, 95,865 children with available data on dental caries, oral hygiene status, and malocclusion were initially selected. After excluding participants with incomplete general characteristics or lifestyle information, a final sample of 93,220 children was included in the main analysis. Because a large proportion of participants lacked periodontal disease data, they were retained in the dataset, and a separate sub-analysis of periodontal disease was conducted using the 16,641 children with available data.

As this study utilized data from a nationwide Student Health Examination, no formal sample size calculation was required, and all eligible participants meeting the inclusion criteria were included in the analysis.

Inclusion criteria: children aged 6–12 years with complete records for the relevant oral health indicators.

Exclusion criteria: participants with missing or incomplete data, particularly those without information on general characteristics or lifestyle variables (except for the separate periodontal disease analysis).

### 2.2. Oral Examination

Students underwent oral examinations by visiting medical institutions that met the qualification standards designated for the national Student Health Examination program. Licensed dentists at these institutions evaluated the overall dental condition, presence and number of decayed teeth (maxillary and mandibular), teeth at risk for developing caries, missing teeth, periodontal condition, and presence of malocclusion. Oral hygiene status was also assessed and classified as good, fair, or requiring improvement.

The following indices were calculated to evaluate oral health status:

- Dental Caries Prevalence (DCP): the proportion of individuals with dental caries among those examined.
- Required Rate of Improved Oral Hygiene (RRIOH): the proportion of individuals assessed as requiring improvement in oral hygiene.
- Periodontal Disease Prevalence (PDP): the proportion of individuals with periodontal disease among those examined.
- Malocclusion Prevalence (MP): the proportion of individuals undergoing or recommended for orthodontic treatment.

Oral diseases were assessed based on dentists’ clinical evaluations.

### 2.3. Demographic Data

The following demographic data were collected from the participants: gender (male or female), city size (metropolitan/small city, town/village, or rural/special area), school grade (elementary grades (first, second, and third), high grades (fourth, fifth, and sixth), and lifestyle factors.

### 2.4. Lifestyle Habits Related to Sleep Duration

Responses to the question about daily sleep duration were divided into four groups. 1: within 6 h, 2: 6–7 h, 3: 7–8 h, and 4: 8 h or more. The four responses were combined to form a reference range for sleep duration based on the sleep hours recommended by the National Sleep Foundation. Short sleep duration was defined as fewer than 8 h, and adequate sleep duration was defined as 8 h or more.

Prolonged media use and exercising three or more times per week were additionally surveyed as lifestyle habit factors related to sleep duration.

Prolonged media use was defined as media use of 2 h or more. Media use included two items: gaming and TV viewing. Each item was evaluated with two responses:

1: 2 h or more and 2: fewer than 2 h.

The question about exercising three or more times per week was also evaluated with two responses:

1: 3 times or more and 2: less than 3 times.

Data were collected from the Subjective Health Survey.

Physical activity levels and sleep duration, which were self-assessed by the school children, were reported to show a high degree of consistency with the objective data and, therefore, could be used effectively as valid indicators of physical activity levels and sleep habits in children in the school environment [25,26].

### 2.5. Ethical Approval

Since only fully anonymized secondary data were used, no direct contact with participants or their guardians occurred, and therefore, informed consent was not applicable. This exemption was confirmed by the Institutional Review Board.

### 2.6. Statistical Analysis

For this study, weighting was applied, and complex sample analysis was conducted. The method for the complex sample analysis performed is as follows.

First, changes in oral health status by year were evaluated using changes in sleep duration and lifestyle habits by year, differences in oral health status according to participants’ sleep duration and lifestyle habits, and differences in sleep duration according to general characteristics and lifestyle habits, and analyzed using the Chi-squared test (Rao-Scott χ^2^ test).

Second, logistic regression analysis was performed to examine the factors affecting oral health and sleep duration.

SPSS Statistics 26 was used for the statistical analyses, and statistical significance was determined at a significance level of 0.05.

## 3. Results

### 3.1. Oral Health Status According to Years

**Figure 1:**
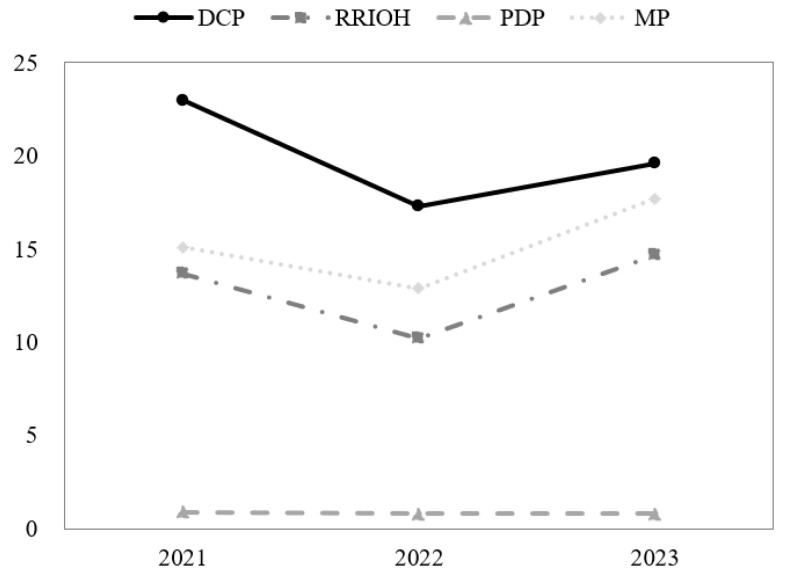
Changes in oral health status over the previous 3 years.

Changes in DCP, PRIOH, PDP, and MP over the past 3 years were evaluated. The three items, DCP, PRIOH, and MP, showed statistically significant changes over the years (*p* < 0.001), with DCP at 23.0% in 2021, 17.3% in 2022, and 19.6% in 2023, showing a decrease in 2022 compared to 2021, followed by a slight increase in 2023. PRIOH was 13.7% in 2021, 10.2% in 2022, and 14.7% in 2023, showing a decrease in 2022 compared to 2021, then an increase in 2023. MP was 15.1% in 2021, 12.9% in 2022, and 17.7% in 2023, showing a decrease in 2022 compared to 2021, then an increase in 2023 (*p* < 0.001). PDP did not show any significant change (*p* < 0.608) (Table 1, Figure 1).

### 3.2. Sleep Duration and Lifestyle According to Year

The changes in sleep duration and lifestyle habits over the past 3 years were evaluated. Sleep duration did not change significantly over the years (*p* = 0.285).

**Figure 2:**
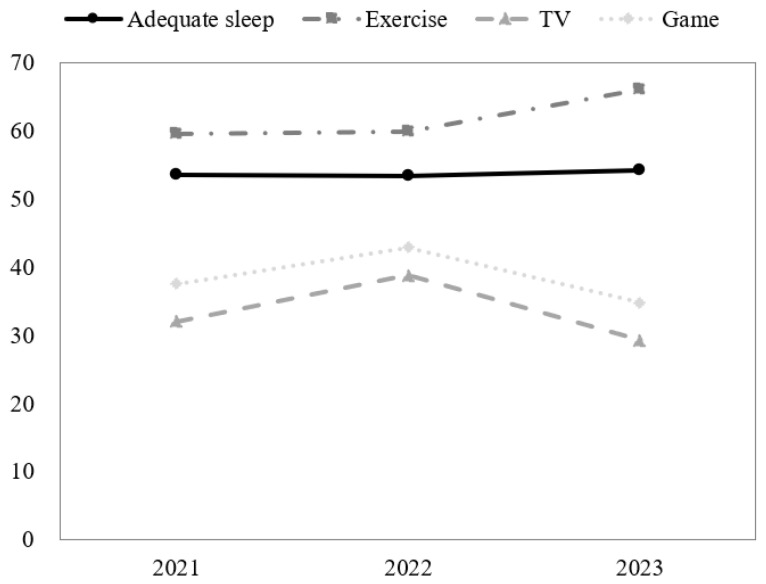
Changes in lifestyle over the previous 3 years.

The three lifestyle habit items showed statistically significant changes over the years (*p* < 0.001). The exercise rate (≥3 times/week) increased from 59.6% in 2021 and from 59.9% in 2022 to 66.1% in 2023. The rate of watching TV for 2 h or more was 32.0% in 2021, 38.8% in 2022, and 29.3% in 2023, showing an increase in 2022 compared to 2021, then a decrease in 2023. The rate of playing games for 2 h or more was 37.5% in 2021, 42.9% in 2022, and 34.8% in 2023, showing an increase in 2022 compared to 2021, then a decrease in 2023 (Table 2, Figure 2).

### 3.3. Oral Health Status According to Sleep Duration and Lifestyle

The differences in DCP, RRIOH, PDP, and MP according to sleep duration and lifestyle habits were evaluated.

DCP was high when sleep duration was short, exercise was not practiced at least 3 times a week, TV was watched for 2 h or more, and games were played for 2 h or more, all of which showed statistically significant differences (*p* < 0.001).

RRIOH was high when sleep duration was short, exercise was not practiced at least 3 times a week, TV was watched for 2 h or more, and games were played for 2 h or more. Statistically significant differences were observed for sleep duration (*p* < 0.001), exercise (*p* < 0.001), TV (*p* = 0.020), and games (*p* < 0.001).

For PDP, the significance probability for sleep duration was slightly higher than 0.05 (*p* = 0.067), but a difference was observed. PDP was relatively high when sleep duration was short.

With a short sleep duration and when TV was watched for less than 2 h, MP was high. Significant differences were observed according to sleep duration (*p* = 0.001) and TV-watching (*p* < 0.001) (Table 3).

### 3.4. Sleep Duration According to Demographic Characteristics and Lifestyle

We analyzed the differences in sleep duration according to demographic characteristics and lifestyle. Significant differences in sleep duration were observed according to city type (*p* = 0.001), school grade (*p* < 0.001), physical activity (*p* < 0.001), watching TV (*p* < 0.001), and gaming (*p* < 0.001). No significant difference was observed by gender (*p* =0.286). In terms of city type, the optimal sleep rate was higher in larger cities. In terms of school grade, the optimal sleep rate was higher in lower school grades. Physical activity three or more times per week, watching TV for fewer than 2 h per day, and gaming for fewer than 2 h per day were associated with a higher optimal sleep rate (Table 4).

### 3.5. Relationship Between Sleep Duration and Lifestyle and Oral Health Status

We analyzed the relationship between sleep duration and lifestyle and DCP, RRIOH, PDP, and MP.

Looking at the factors affecting DCP, the probability of dental decay was significantly higher in those with short sleep durations (odds ratio (OR) = 1.14, *p* < 0.001). For those who performed physical activity less than three times per week, the probability of DCP was significantly higher (OR = 1.10, *p* = 0.001), and for those who watched TV for more than 2 h, the probability of DCP was also significantly higher (OR = 1.06, *p* = 0.038). Similarly, the probability of DCP was significantly higher for those who played games for more than 2 h (OR = 1.22, *p* < 0.001).

Looking at the factors affecting RRIOH, the probability of needing oral hygiene improvement was significantly higher in those who performed physical activity less than three times per week (OR = 1.18, *p* < 0.001), and for those who played games for more than 2 h, the probability of PRIOH was significantly higher (OR = 1.16, *p* < 0.001). Although the OR for sleep duration was slightly higher than 0.05, in the case of short sleep duration, the probability of PRIOH was higher (OR = 1.06, *p* = 0.055).

Looking at the factors affecting PDP, a significant result was observed for sleep duration, with the probability of periodontal disease being significantly higher in those with short sleep duration (OR = 1.64, *p* = 0.045).

Looking at the factors affecting the probability of malocclusion, watching TV (OR = 0.88, *p* < 0.001) and gaming (OR = 0.94, *p* = 0.031) for more than 2 h were associated with a significantly lower probability of malocclusion.

In summary, short sleep duration increases the probability of dental decay, the need for oral hygiene improvement, and the probability of periodontal disease.

For those who performed physical activity less than three times per week, the probability of dental decay and the need for oral hygiene improvement increased. For those who watched TV for more than 2 h, the probability of dental decay increased, and the probability of malocclusion decreased. The probability of dental decay and the need for oral hygiene improvement increased for those who played games for more than 2 h, while the probability of malocclusion decreased (Table 5).

### 3.6. Relationship Between Demographic Characteristics, Lifestyle, and Sleep Duration

The relationship between demographic data, lifestyle habits, and sleep duration was evaluated. Compared to rural areas, metropolis (city: OR = 1.09, *p* = 0.038) and urban districts (OR = 1.13, *p* = 0.025) showed significantly higher probabilities of receiving optimal sleep, whereas the probability of receiving optimal sleep was significantly lower in higher grades compared to lower grades (OR = 0.40, *p* < 0.001). For exercise, those who exercised three or more times per week had a significantly higher probability of getting optimal sleep (OR = 1.26, *p* < 0.001), whereas watching TV for more than 2 h (OR = 0.86, *p* < 0.001) and playing games for more than 2 h (OR = 0.58, *p* < 0.001) were associated with a significantly lower probability of getting optimal sleep (Table 6).

## 4. Discussion

This study analyzed the effects of sleep habits and lifestyle habits (exercise and media use) of elementary school students on oral health. Sleep deprivation, exercise, and media use had significant effects on the Incidence rate of dental caries (DCP), the need for oral hygiene improvement (RRIOH), PDP, and MP, consistent with the results of several previous studies.

### 4.1. Relationship Between Short Sleep, Exercise Frequency, and Media Use with Oral Health

Several previous studies have observed a relationship between sleep duration and oral health [27,28,29]. Consistent with the results of this study, a population-based cohort study conducted in Japan showed that the shorter the sleep duration, the higher the Incidence rate of dental decay, indicating that sleep in adolescence is an important protective factor in preventing dental decay [27].

Recent studies have focused not only on dental caries but also on the relationship between periodontal status and sleep habits. According to studies such as that by Mehdipour et al., although no statistically significant association between sleep deprivation and dental caries in children was found, the prevalence of periodontitis was higher, which may be related to decreased immune function and increased inflammatory response in the oral cavity due to sleep deprivation [28]. In this study, short sleep duration was associated with a higher prevalence of dental caries and PDP, which is both consistent and inconsistent with previous findings.

Also, the association between short sleep duration, prolonged media use, and dental caries observed in this study is consistent with the results of studies such as that by Asaka et al. In that study, children with fewer than 8 h of sleep and more than 2 h of media use showed a higher prevalence of dental decay, suggesting that irregular lifestyle habits may negatively affect oral health [29].

Unlike expectations regarding malocclusion and media use, when TV and game time exceeded 2 h, MP was observed to decrease, which may be due to changes in oral habits (e.g., tongue position and the use of oral muscles) caused by prolonged media use. Therefore, additional research is needed.

### 4.2. Analysis of Demographic Factors

Differences in oral health and sleep duration according to city type, grade, and gender were confirmed in this study.

Examining the differences in sleep duration according to demographic data, significant differences in sleep duration were observed according to city type (*p* = 0.001), grade (*p* < 0.001), exercising (*p* < 0.001), watching TV (*p* < 0.001), and playing games (*p* < 0.001). No significant difference was observed according to gender (*p* = 0.286). The larger the city size and the lower the grade level, the higher the likelihood of obtaining adequate sleep.

Looking at the factors affecting sleep duration, children living in metropolis (OR = 1.09, *p* = 0.038) and urban districts (OR = 1.13, *p* = 0.025) had a significantly higher probability of obtaining adequate sleep compared with those in rural areas, whereas students in higher grades were significantly less likely to obtain adequate sleep than those in lower grades (OR = 0.40, *p* < 0.001)

This finding aligns with previous literature examining the role of environmental and developmental factors in children’s sleep. For example, Mayne et al. investigated neighborhood environments and children’s sleep and found that more favorable urban neighborhood characteristics—such as infrastructure, lighting, and environmental regulation—were associated with better sleep outcomes, including longer sleep duration and improved sleep quality [30]. These results suggest that urban contexts may, under certain conditions, provide more supportive environments for children’s sleep. In addition, Yip et al. reported that younger children obtained significantly longer sleep durations compared with older children, supporting the idea that grade level is a strong determinant of sleep adequacy [31]. Taken together, these findings corroborate our results that urban residency and lower grade levels are linked to higher probabilities of obtaining sufficient sleep, highlighting the complex interplay between environmental and developmental factors in shaping sleep behaviors among children.

Looking at the factors affecting DCP, the DCP of boys was significantly lower (OR = 0.90, *p* < 0.001), and the DCP of higher grades was significantly lower (OR = 0.89, *p* < 0.001). This finding is consistent with several previous reports indicating that female children and adolescents often experience a higher caries burden than males. Lukacs and Largaespada suggested that biological factors such as earlier tooth eruption in girls, hormonal changes during puberty, and sex differences in salivary composition may contribute to this disparity [32]. Similarly, a school-based cross-sectional study demonstrated that female students had significantly higher DMFT scores compared with males, highlighting greater caries susceptibility among girls in certain settings [33]. Research conducted in Saudi Arabia further supported these findings, reporting that female adolescents had a higher prevalence of decayed, missing, and filled teeth compared to their male counterparts [34].

Looking at the factors affecting the need for oral hygiene improvement, the OR was significantly higher in urban areas (OR = 1.17, *p* = 0.014) and suburban areas (OR = 1.27, *p* = 0.001) compared to rural areas, and males had a significantly higher RRIOH than females (OR = 1.14, *p* < 0.001), and high school students had a significantly higher RRIOH than middle school students (OR = 1.51, *p* < 0.001). These patterns are consistent with the literature, indicating that men generally demonstrate poorer oral hygiene practices and less preventive care uptake compared with women (e.g., less frequent brushing, flossing, and dental visits) [35]. The regional effect parallels studies showing that rural populations often face greater barriers to dental services and lower preventive care utilization, which might paradoxically lower observed “need” for hygiene improvement (due to under-detection) but worsen disease burden in the long term [36].

Looking at the factors affecting MP, the OR for MP in urban areas was significantly higher than in rural areas (OR = 1.28, *p* < 0.001), the MP of males was significantly lower than that of females (OR = 0.91, *p* = 0.001), and the MP was significantly higher in higher grades (OR = 1.52, *p* < 0.001). While these findings suggest that residence, gender, and grade level are important determinants of malocclusion prevalence, evidence from previous studies has not always been consistent. For example, Shen et al. found no significant differences in malocclusion prevalence by sex or urban–rural residence in younger children [37]. Additionally, Alhammadi et al. similarly reported that global studies show wide variability without consistent demographic associations [38].

### 4.3. COVID-19 Pandemic and Endemic Correlation

In 2021~2022, due to the impact of COVID-19, social distancing and remote classes continued, which affected the lifestyle habits of children. During the pandemic period, the prevalence of dental decay and the rate of needing oral hygiene improvement decreased, but after 2023, an increasing trend was observed again. This can be explained by the following hypotheses.

First, during the pandemic, as eating out and dental visits decreased and home meals increased, refined sugar intake decreased, which may have temporarily reduced dental decay. In fact, a study by Akşit-Bıçak also reported that the consumption of such foods decreased during the pandemic period [39]. Second, due to increased awareness of mask-wearing and hygiene, handwashing habits may have been strengthened. Some studies have reported that the frequency of health management at home increased during the pandemic period [40]. However, after the end of pandemic, with increased eating out, weakened hygiene habits, and increased academic stress, there is a possibility that oral health deteriorated again.

Also, during the pandemic, media use time continued to increase but decreased in 2023, while the rate of physical activity increased significantly in 2023. This may be related to the normalization of school classes and activities and suggests that overall life rhythm recovery is underway.

### 4.4. Strengths and Limitations of the Study

The strength of this study is that it analyzed the impact of various demographic and lifestyle factors on oral health using a large-scale sample of 93,220 participants. However, this study was based on a cross-sectional study design, so it is limited in establishing causality, and since sleep time and lifestyle habits were collected by self-reported methods, there is a possibility of some bias. Also, since the number of participants analyzed for PDP was relatively small, caution is needed when interpreting the results. In this study, only sleep time was evaluated, and the quality of sleep was not analyzed. According to the definition of optimal sleep as 8 h or more, excessive sleep of 12 h or more was also included as optimal sleep. This point requires caution when interpreting sleep-related variables.

### 4.5. Future Research Directions

In future research, it is necessary to clearly analyze the causal relationship between sleep and oral health through longitudinal studies and to introduce objective sleep assessment tools such as wearable devices. Research is also needed to comprehensively analyze various lifestyle factors, such as media use, eating habits, and emotional factors.

## 5. Conclusions

According to our research results, DCP, PRIOH, and MP decreased in 2022 compared to 2021 but increased again in 2023. The lifestyle habits that affected oral health included sleep duration, exercise frequency, and media use. There was a significant correlation between regional health status and sleep time, and the shorter the sleep time, the worse the regional health status. The lifestyle habits of physical activity and media use were also related to children’s regional health and affected sleep time. This suggests that sleep time is an important factor in improving children’s regional health. Therefore, sufficient sleep, regular physical activity, and restricted media use are emphasized for the prevention of oral disease.

## Figures and Tables

**Table 1:** Changes in oral health status over the previous 3 years.

Variables	2021 (n = 34,203)	2022 (n = 34,626)	2023 (n = 24,391)	*p*-Value
DCP	7563 (23.0)	6450 (17.3)	4706 (19.6)	<0.001
RRIOH	4283 (13.7)	4142 (10.2)	3150 (14.7)	<0.001
PDP *	75 (0.9)	68 (0.8)	39 (0.8)	0.608
MP	4838 (15.1)	5058 (12.9)	3908 (17.7)	<0.001

Values are presented as number (weighted %); * n (2021) = 6522, n (2022) = 5584, n (2023) = 4535.

**Table 2:** Changes in sleep duration and lifestyle over the previous 3 years.

Variables	2021 (n = 34,203)	2022 (n = 34,626)	2023 (n = 24,391)	*p*-Value
Adequate sleep	18,277 (53.6)	18,638 (53.4)	13,369 (54.2)	0.285
Exercise	20,214 (59.6)	21,363 (59.9)	15,916 (66.1)	<0.001
TV	11,307 (32.0)	11,060 (38.8)	7350 (29.3)	<0.001
Games	13,103 (37.5)	13,259 (42.9)	8993 (34.8)	<0.001

**Table 3:** DCP, RRIOH, PDP, MP according to sleep duration and lifestyle.

Variables	Categories	DCP	RRIOH	PDP	MP
Sleep duration	Adequate (≥8 h)	9504 (20.2)	5677 (13.0)	70 (0.7)	7128 (15.7)
	Short (<8 h)	9215 (22.7)	5898 (15.4)	112 (1.1)	6676 (17.0)
	*p*-value	<0.001	<0.001	0.067	0.001
Exercise	≥3 times	11,114 (20.5)	6800 (13.2)	115 (0.8)	8438 (16.2)
	<3 times	7605 (22.8)	4775 (15.5)	67 (1.0)	5366 (16.5)
	*p*-value	<0.001	<0.001	0.447	0.430
TV	≥2 h	6370 (22.9)	3913 (14.7)	51 (0.8)	4156 (14.8)
	<2 h	12,349 (20.7)	7662 (13.8)	131 (0.9)	9648 (16.9)
	*p*-value	<0.001	0.020	0.578	<0.001
Games	≥2 h	7930 (23.8)	5175 (16.3)	73 (0.9)	5407 (16.1)
	<2 h	10,789 (20.0)	6400 (12.9)	109 (0.8)	8397 (16.4)
	*p*-value	<0.001	<0.001	0.656	0.557

Values are presented as numbers (weighted %). Total samples of DCP, RRIOH, MP: n = 93,220; Total samples of PDP: n = 16,641.

**Table 4:** Sleep duration according to general characteristics and lifestyle.

Variables	Categories	Sleep Duration		*p*-Value
		Adequate (≥8 h)	Short (<8 h)	
City type	Metropolis	40,602 (54.2)	34,175 (45.8)	0.001
	Urban	5990 (53.1)	5560 (46.9)	
	Rural	3692 (50.6)	3201 (49.4)	
Sex	Male	26,019 (54.1)	21,767 (45.9)	0.286
	Female	24,265 (53.6)	21,169 (46.4)	
School grade	High grade (4~6)	19,642 (41.6)	27,592 (58.4)	<0.001
	Low grade (1~3)	30,642 (66.6)	15,344 (33.4)	
Exercise	≥3 times	32,655 (56.9)	24,838 (43.1)	<0.001
	<3 times	17,629 (48.7)	18,098 (51.3)	
TV	≥2 h	14,685 (48.9)	15,032 (51.1)	<0.001
	<2 h	35,599 (56.1)	27,904 (43.9)	
Games	≥2 h	14,799 (41.5)	20,556 (58.5)	<0.001
	<2 h	35,485 (60.9)	22,380 (39.1)	

Values are presented as numbers (weighted %).

**Table 5:** Relationship between sleep duration, lifestyle, and oral health status.

Variables	Categories	DCP		RRIOH		PDP		MP	
		OR (95% CI)	*p*-Value	OR (95% CI)	*p*-Value	OR (95% CI)	*p*-Value	OR (95% CI)	*p*-Value
Sleep duration	Short	1.14 (1.08~1.20)	<0.001	1.06 (1.00~1.13)	0.055	1.64 (1.01~2.67)	0.045	1.01 (0.95~1.07)	0.698
	Adequate	(reference)		(reference)		(reference)		(reference)	
Exercise	<3 times	1.10 (1.04~1.15)	0.001	1.18 (1.11~1.25)	<0.001	1.25 (0.77~2.02)	0.373	1.00 (0.95~1.06)	0.872
	≥3 times	(reference)		(reference)		(reference)		(reference)	
TV	≥2 h	1.06 (1.00~1.12)	0.038	1.01 (0.95~1.08)	0.698	0.79 (0.44~1.40)	0.413	0.88 (0.83~0.94)	<0.001
	<2 h	(reference)		(reference)		(reference)		(reference)	
Game	≥2 h	1.22 (1.16~1.29)	<0.001	1.16 (1.09~1.24)	<0.001	1.12 (0.68~1.83)	0.665	0.94 (0.88~0.99)	0.031
	<2 h	(reference)		(reference)		(reference)		(reference)	

**Table 6:** Relationship between demographic characteristics, lifestyle, and sleep duration.

Variables	Categories	DCP		RRIOH		PDP		MP	
		OR(95% CI)	*p*-Value	OR(95% CI)	*p*-Value	OR(95% CI)	*p*-Value	OR(95% CI)	*p*-Value
City type	Metropolis	0.96 (0.86~1.06)	0.388	1.17 (1.03~1.33)	0.014	4.11 (0.99~17.01)	0.051	1.28 (1.15~1.42)	<0.001
	Urban	1.12 (0.99~1.26)	0.066	1.27 (1.10~1.47)	0.001	2.72 (0.61~12.12)	0.189	1.05 (0.91~1.20)	0.505
	Rural	(reference)		(reference)		(reference)		(reference)	
Sex	Male	0.90 (0.86~0.95)	<0.001	1.14 (1.07~1.21)	<0.001	1.22 (0.76~1.97)	0.415	0.91 (0.86~0.96)	0.001
	Female	(reference)		(reference)		(reference)		(reference)	
School grade	High grade (4~6)	0.89 (0.85~0.94)	<0.001	1.51 (1.41~1.61)	<0.001	0.80 (0.50~1.27)	0.341	1.52 (1.43~1.61)	<0.001
	Low grade (1~3)	(reference)		(reference)		(reference)		(reference)	

## Data Availability

The data are not publicly available because they can only be accessed through the official website of the Student Health Information Center, which requires a data request and consent for the collection and use of personal information.
